# Dietary Flavonoids Alleviate Inflammation and Vascular Endothelial Barrier Dysfunction Induced by Advanced Glycation End Products In Vitro

**DOI:** 10.3390/nu14051026

**Published:** 2022-02-28

**Authors:** Yishan Fu, Yijia Jia, Yilin Sun, Xiaojing Liu, Junjie Yi, Shengbao Cai

**Affiliations:** Faculty of Food Science and Engineering, Kunming University of Science and Technology, Kunming 650500, China; fuyishankm@163.com (Y.F.); yijia529@163.com (Y.J.); syl115630519@163.com (Y.S.); xiaojingliu_kmust@163.com (X.L.); junjieyi@kust.edu.cn (J.Y.)

**Keywords:** advanced glycation end products, dietary flavonoids, endothelial dysfunction, inflammation, molecular docking

## Abstract

The aim of this study was to compare the protective effects of three dietary flavonoids (apigenin-7-*O*-glucoside (A7G), isorhamnetin-3-*O*-rutinoside (I3R), and cyanidin-3-*O*-glucoside (C3G)) on advanced glycation end products (AGEs)-induced inflammation and vascular endothelial dysfunction. Furthermore, the potential mechanisms of varied effects of those three dietary flavonoids were analyzed by molecular docking analysis. Results showed that C3G (40 μM) achieved the best inhibition on inflammatory cytokines (TNF-α, IL-1β, and IL-6) in AGEs-induced RAW264.7 cells, followed by I3R, and A7G was the weakest. The molecular docking results also showed that C3G exhibited the closest binding with the receptor for AGE. However, I3R (40 μM) demonstrated the best effect in improving endothelial dysfunction in AGEs-induced EA.hy926 cells, followed by C3G, and A7G was the weakest, as evidenced by the molecular docking results of flavonoids with profilin-1. This work may provide knowledge and helpful suggestions regarding the benefits of dietary flavonoids in diabetic vascular complications.

## 1. Introduction

Type 2 diabetes mellitus (T2DM) is a serious and common global disease that affects more than 360 million people worldwide [1]. The number of individuals with T2DM is still increasing and is expected to reach 693 million cases by 2045 [1]. Vascular complications are considered one of the leading causes of death from diabetes. According to previous reports, 50% of patients with diabetes die every year from vascular complications [2,3]. During T2DM, long-term hyperglycemia leads to the production and accumulation of advanced glycation end products (AGEs) in blood [4]. Studies have reported that AGEs are believed to be key factors that promote vascular damage and inflammation through receptor-independent and receptor-dependent mechanisms, leading to a series of vascular complications [5,6]. The combination of AGEs and their receptors (i.e., RAGE, receptor for AGEs) can activate macrophages and produce excessive pro-inflammatory cytokines through RAGE, such as tumor necrosis factor-α (TNF-α), interleukin-1β (IL-1β), and interleukin-6 (IL-6) [4]. In addition, AGEs can also induce endothelial cell damage through profilin-1 (PFN1), causing the reorganization and redistribution of endothelial cytoskeleton actin and leading to endothelial cell dysfunction [6]. Moreover, the overproduction of reactive oxygen species (ROS) caused by AGEs is also considered a trigger for inflammation and vascular endothelial (VE)-cadherin endocytosis associated with the maintenance of the cytoskeleton [4,7]. Therefore, finding nontoxic and high-efficiency dietary bioactive compounds to improve AGEs-induced inflammation and endothelial dysfunction may be an effective strategy for preventing or alleviating chronic diseases caused by the vascular complications of diabetes.

Dietary polyphenols (e.g., phenolic acids, flavonoids, and anthocyanins) comprise one type of the most important secondary metabolites in edible plants. They have been proven to exhibit a variety of biological activities and play important roles in protecting human health and preventing vascular complications [8]. Several studies have shown that dietary polyphenols can improve inflammation and oxidative damage induced by AGEs [9,10]. Teng et al. reported that naringenin significantly reduced the generation of ROS induced by AGEs and the content of some inflammatory mediators in RAW264.7 cells [9]. Yu et al. found that the polyphenolic substance pterostilbene in blueberries can inhibit AGEs-induced oxidative stress and inflammation [10]. Moreover, white wine pomace products (rich in polyphenols) have been found to improve endothelial dysfunction and prevent endothelial injury under hyperglycemic conditions [7]. Loke et al. determined that quercetin (dietary flavonoids) reduced atherosclerosis in mice by improving endothelial dysfunction [11]. However, only a few studies have comparatively investigated the effects and mechanisms of different dietary flavonoids on AGEs-induced inflammation and vascular endothelial dysfunction.

In our previous studies, we found that apigenin-7-*O*-glucoside (A7G), isorhamnetin-3-*O*-rutinoside (I3R), and cyanidin-3-*O*-glucoside (C3G) were the three most effective flavonoids for inhibiting the activities of α-glucosidase and dipeptidyl peptidase-IV and improving insulin resistance among more than 20 dietary flavonoids that may be beneficial for diabetic patients in controlling blood glucose [12,13]. However, information about the effects and mechanisms of the three flavonoids on AGEs-induced inflammation and vascular endothelial dysfunction remain unknown. Such information may facilitate the further understanding and utilization of dietary flavonoids for improving the condition of diabetic patients. Therefore, the differences in the effects of the three dietary flavonoids (i.e., A7G, I3R, and C3G) on AGEs-induced inflammation and endothelial dysfunction were comparatively explored in the current study via cell experiments. Moreover, the potential reasons why those three dietary flavonoids possessed different effects were analyzed by molecular docking analysis. The results of this study, combined with our previous findings, may provide a systematic knowledge and suggestion of dietary flavonoids for diabetic patients to improve their conditions.

## 2. Materials and Methods

### 2.1. Materials and Reagents

Dulbecco’s modified Eagle’s medium (DMEM) and fetal bovine serum (FBS) were purchased from Gibco (Grand Island, NY, USA). Penicillin–streptomycin and 0.25% trypsin–EDTA solution were obtained from Solarbio (Beijing, China). Bovine serum albumin (BSA) was procured from Biorigin (Beijing, China). The antibody of VE-cadherin (A0734) was acquired from ABclonal (Wuhan, China). NO test kits (S0021S) were procured from Beyotime Biotechnology Co., Ltd. (Shanghai, China). A7G, I3R, and C3G (purity: ≥95.0%) were purchased from Chengdu Must Biotechnology Co., Ltd. (Chengdu, China). Other chemicals and solvents were of analytical grade.

### 2.2. Preparation of AGEs

AGEs were prepared through the reaction of fructose and BSA in accordance with the methods reported by Zeng with minor modifications [14]. First, 1.5 g of BSA was dissolved in 50 mL of phosphate-buffered saline (PBS; pH 7.4, 0.2 M), and then the BSA solution (30 mg/mL) was incubated with or without 500 mM fructose for 60 days at 37 °C. After incubation, the reaction mixture was placed in a 10 kD dialysis bag and dialyzed in PBS (pH 7.4, 0.2 M) at 4 °C for 24 h. Thereafter, the AGEs were lyophilized and stored at −20 °C until use. Considering that AGEs exhibit self-fluorescence, the fluorescence value of their reaction mixture was measured to determine AGE production [14]. In the current study, the fluorescence value was measured at 370 nm excitation and 440 nm emission wavelengths by using a SpectraMax M5 microplate (Molecular Device, Sunnyvale, CA, USA) (Figure S1). The fluorescence value of the AGE solution (equivalent to 1.0 mg/mL of BSA, 5572.23 ± 19.95) was significantly higher than that of the control group (1.0 mg/mL of BSA, 77.77 ± 2.99; *p* < 0.05), indicating that the AGEs were prepared well.

### 2.3. Effects of Dietary Flavonoids on AGEs-Induced ROS Formation in RAW264.7 and EA.hy926 Cells

RAW264.7 macrophage cells and EA.hy926 endothelial cells were obtained from the cell bank of the Chinese Academy of Sciences (Kunming, China) and cultured in DMEM with 10% FBS and 1% penicillin–streptomycin at 37 °C, 5% CO_2_. Before the formal experiment, the MTT method was used to determine the appropriate concentrations of flavonoids and AGEs. The effects of flavonoids on ROS levels in the two cell lines induced by AGEs were investigated following the method of Teng et al. with minor modifications [9]. Cells (1 × 10^5^ cells/mL) were seeded into a six-well plate at 2.0 mL per well. After being cultured for 24 h, RAW264.7 cells were treated with a medium containing 200 μg/mL of BSA or AGEs (with or without addition flavonoids), and EA.hy926 cells were treated with a medium containing 800 μg/mL BSA or AGEs (with or without addition flavonoids). The wells containing only BSA were the control group, the wells containing only AGEs were the model group, and the wells containing the mixture of AGEs and flavonoids were the sample groups. After being incubated for another 24 h, the cells were washed two times with PBS and incubated with 10 μmol/L of dichlorodihydrofluorescein diacetate for 20 min in the dark at 37 °C. Thereafter, the cells were washed two times with an FBS-free medium to prepare the cell suspension, and ROS production was detected using a guava^®^ easyCyte™ 6-2L flow cytometer (Millipore, Billerica, MA, USA).

### 2.4. Effects of Dietary Flavonoids on the Secretion of Inflammatory Cytokines in AGEs-Induced RAW264.7 Cells

RAW264.7 cells (1 × 10^5^ cells/mL) were seeded into a six-well plate at 2.0 mL per well for 24 h and then treated with a medium that contained 200 μg/mL of BSA or AGEs (with or without addition flavonoids) for another 24 h. Thereafter, the cell supernatants were collected for inflammatory cytokine tests [4]. The levels of inflammatory cytokines, including IL-1β, IL-6, and TNF-α, were measured using the corresponding enzyme-linked immunosorbent assay (ELISA) kits in accordance with their instructions.

### 2.5. Effects of Dietary Flavonoids on NO Production in AGEs-Induced EA.hy926 Cells

EA.hy926 cells (1 × 10^5^ cells/mL) were seeded into a 96-well plate at 200 μL per well for 24 h and then incubated with 800 μg/mL of BSA or AGEs with or without addition flavonoids for another 24 h. Thereafter, an NO test kit was used to determine cell supernatants in accordance with the instructions. Cell viability in each corresponding well determined via MTT assays was used to normalize the cellular production of NO [7].

### 2.6. Immunofluorescence Analysis

The immunofluorescence of VE-cadherin was determined by referring to a previous study with slight modifications [7]. EA.hy926 cells (1 × 10^5^ cells/mL) were seeded into a six-well plate at 2 mL per well for 24 h and cultured with 800 μg/mL BSA or AGEs (with or without addition flavonoids) for another 24 h. Thereafter, the cells were placed onto slides, fixed with 4% paraformaldehyde, infiltrated with 0.05% Triton X-100, incubated with the corresponding antibody (VE-cadherin), and stained. The nuclei were stained with DAPI. All the slides were washed and observed under an Olympus IX83 microscope (Tokyo, Japan) at 200 × magnification. Image analysis was performed via ImageJ software (National Institutes of Health, Bethesda, Maryland, USA).

### 2.7. Transepithelial Electrical Resistance (TEER) Measurement

TEER measurements can be used to check intercellular integrity and permeability. To detect TEER value, the method reported by Wu et al. was used with several modifications [15]. EA.hy926 cells (1 × 10^5^ cells/mL) were seeded into a chamber of a Transwell 12-well plate at 500 μL per well. After 24 h of cultivation, BAS or AGE medium (with or without addition flavonoids) was added. Resistance values were detected every 12 h by using a Millicell-ERS-2 voltmeter (Millipore Continental Water Systems, Bedford, MA, USA).

### 2.8. Molecular Docking Analysis

The binding properties between RAGE (PDB Code: 3CJJ) and three dietary flavonoids and between PNF1 (PDB Code: 3NUL) and three dietary flavonoids were investigated via Sybyl-x2.1.1 software [12]. The structures of RAGE and PNF1 were downloaded from the Research Collaboratory for Structural Bioinformatics protein database. Before docking, all the water molecules and other residues were removed, and AMBER7-FF99 charges and hydrogen atoms were added for structure preparation. T-score and C-score calculations were applied to determine superior analysis results. Ligplot^+^ software was used to analyze hydrophobic interactions.

### 2.9. Statistical Analysis

All the experiments were performed at least three times, and the results are expressed as mean ± SD. One-way ANOVA and Tukey’s test were conducted to evaluate the significant differences (*p* < 0.05) by using Origin 8.5 software (OriginLab, Northampton, MA, USA).

## 3. Results and Discussion

### 3.1. Dietary Flavonoids Suppressed AGEs-Induced ROS Production

Oxidative stress is defined as the excessive production of cellular ROS, including superoxide anion and hydrogen peroxide [16]. Excessive ROS caused by exogenous substances, such as AGEs, can disrupt the balance of cell redox [1]. Under pathological conditions, ROS produces oxidative stress that not only causes cellular inflammation but also vascular endothelial dysfunction and an increase in vascular endothelial permeability, causing irreversible damage to cellular structures and functions and leading to vascular complications [1,17].

**Figure 1:**
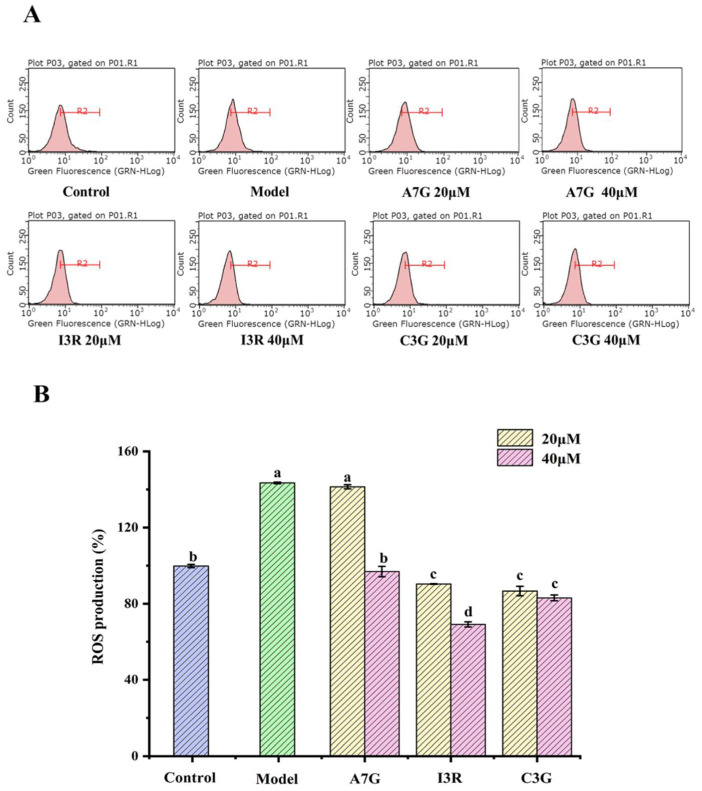
Inhibitory effects of apigenin-7-*O*-glucoside (A7G), isorhamnetin-3-*O*-rutinoside (I3R), and cyanidin-3-*O*-glucoside (C3G) on AGEs-induced RAW264.7 cells cellular ROS. (**A**) Flow cytometer analysis. (**B**) Relative content of ROS among different groups. The values are presented as mean ± SD of three replicates. Different letters (a, b, c, d) indicate significant differences (*p* < 0.05).

**Figure 2:**
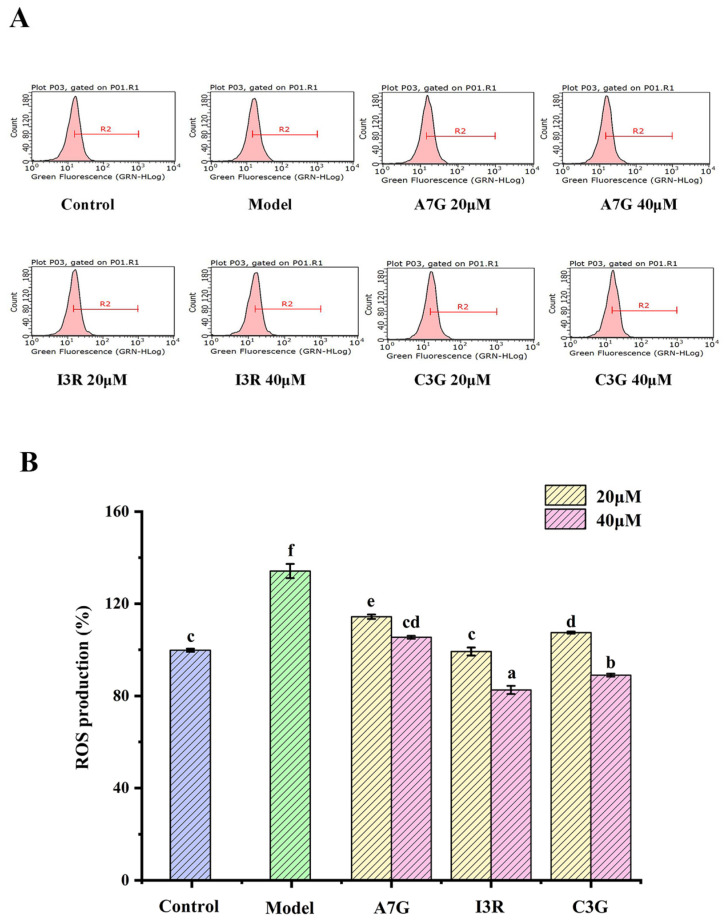
Effects of A7G, I3R, and C3G on AGEs-induced EA.hy926 cellular ROS inhibition of cells. (**A**) Flow cytometer analysis. (**B**) Relative content of ROS among different groups. Values are expressed as mean ± SD of three replicates. Different letters (a, b, c, d, e, f) indicate significant differences (*p* < 0.05).

In the current study, the effects of three dietary flavonoids on ROS production in AGEs-induced RAW264.7 and EA.hy926 cells were investigated. As shown in Figure 1, the ROS content of the RAW264.7 cells in the model group (treated with 200 μg/mL AGEs) was significantly higher than that of the control group (treated with 200 μg/mL BSA) (*p* < 0.05), and the ROS content of the model group reached as high as 143.65% ± 0.41%. Similarly, the ROS content of the EA.hy926 cells in the model group (treated with 800 μg/mL AGEs) was significantly higher than that of the control group (treated with 800 μg/mL BSA) (*p* < 0.05), as shown in Figure 2, and the relative ROS content of the model group was 134.42% ± 3.10%. These results suggest that AGE treatment can increase oxidative stress in both types of cells. Compared with the model group, I3R and C3G significantly inhibited ROS production in RAW264.7 and EA.hy926 cells at concentrations of 20 μM and 40 μM (*p* < 0.05). I3R at a concentration of 40 μM exhibited the strongest inhibition against ROS production in both cells (RAW264.7 cells, producing 69.14% ± 1.33%; EA.hy926 cells, producing 82.61% ± 1.78%). Compared with the two other dietary flavonoids, however, A7G exhibited the weakest inhibitory effect on ROS production in both cells (*p* < 0.05), and the relative ROS production in AGEs-induced RAW264.7 cells with 20 μM treatment was even similar to that in the model group (*p* > 0.05).

The preceding results clearly demonstrated that A7G, I3R, and C3G can inhibit AGEs-induced intracellular oxidative stress in RAW264.7 and EA.hy926 cells to different degrees. Such capability may be beneficial for cellular inflammation and endothelial dysfunction caused by AGEs. A previous study reported that when AGEs combined with RAGE, various intracellular signal transductions were activated to promote ROS production through the nicotinamide adenine dinucleotide phosphate oxidation system, resulting in an oxidative environment in the body and subsequently triggering inflammation [18]. Ferulic acid, a dietary phenolic compound, has been confirmed to inhibit AGEs-induced inflammatory response by mitigating oxidative stress in human umbilical vein endothelial cells [4]. Yu et al. reported that 4′-methoxyresveratrol significantly reduced ROS production and protected against oxidative stress damage to alleviate AGEs-induced inflammation in RAW264.7 cells [19]. Teng et al. demonstrated that naringin alleviated oxidative stress to suppress inflammation in AGEs-induced RAW264.7 cells [9]. Moreover, a previous study found that when the oxidative stress level of endothelial cells increased, the biological barrier formed by endothelial cells between blood flows and surrounding tissues will change to a certain extent, and consequently, the changed endothelial function will lead to endothelial dysfunction [7]. Loke et al. determined that quercetin can reduce oxidative stress to improve endothelial function in rats [11].

### 3.2. Dietary Flavonoids Reduced Pro-Inflammatory Cytokines Secretion in AGEs-Induced RAW264.7 Cells

**Figure 3:**
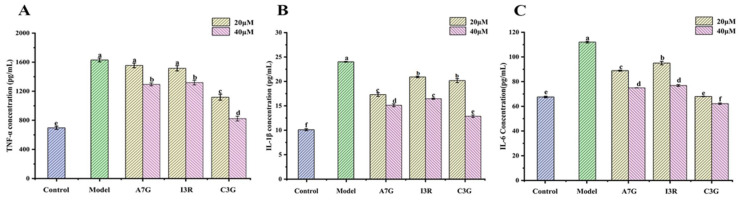
Effects of A7G, I3R, and C3G on three pro-inflammatory cytokines, including TNF-α (**A**), IL-1β (**B**), and IL-6 (**C**) in RAW264.7 cells induced by AGEs. Different letters (a, b, c, d, e, f) indicate significant differences (*p* < 0.05).

The accumulation of AGEs can not only increase the production of ROS but also stimulate macrophages to produce excessive pro-inflammatory cytokines. TNF-α, IL-1β, and IL-6 are typically considered biomarkers of inflammation induced by AGEs [4]. Thus, the levels of IL-1β, IL-6, and TNF-α were measured via ELISA in the present work to assess the effects of the three dietary flavonoids on the inflammatory response of AGEs-induced RAW264.7 cells. As shown in Figure 3, the expression levels of TNF-α, IL-1β, and IL-6 in the model group (treated with 200 μg/mL AGEs) were significantly higher than those in the control group (treated with 200 μg/mL BSA; *p* < 0.05), increasing by 2.36, 2.37, and 1.66 times, respectively. However, compared with the model group, C3G (40 μM) exhibited the strongest inhibitory effect on the expression of inflammatory cytokines (*p* < 0.05), and the expression of TNF-α, IL-1β, and IL-6 decreased by 1.97, 1.86, and 1.80 times, respectively. I3R and A7G also demonstrated good inhibitory effects on the expression of the three inflammatory cytokines, particularly at 40 μM. Singh et al. indicated that high concentrations of TNF-α can disrupt the immune balance of the body and interact with other pro-inflammatory factors, such as IL-1β and IL-6, to cause inflammatory damage [20]. Mirza found that a continuously high level of IL-1β is an important cause of severe wound inflammation among diabetic patients [21]. The results of the current study suggested that the three dietary flavonoids may reduce AGEs-induced inflammation by inhibiting the expression of inflammatory cytokines. This finding is consistent with that of a previous report that naringin reduces AGEs-induced TNF-α, COX-2, and IL-1β expression [9].

### 3.3. Protective Effects of Dietary Flavonoids on Endothelial Barrier Function in AGEs-Induced EA.hy926 Cells

**Figure 4:**
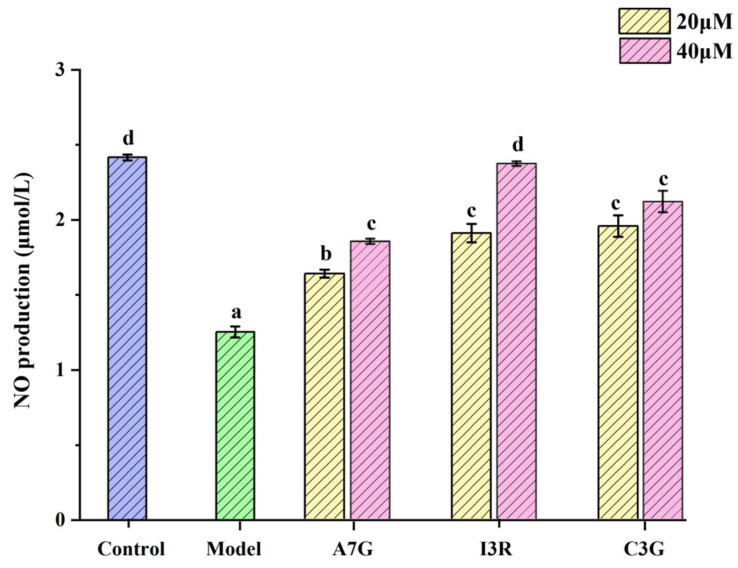
NO production by EA.hy926 cells by A7G, I3R, and C3G. Values are expressed as mean ± SD of three replicates. Different letters (a, b, c, d) indicate significant differences (*p* < 0.05).

One of the most apparent characteristics of endothelial dysfunction is the increase in endothelial permeability due to changes in adhesion cells, allowing macromolecules to pass through the barrier of vascular endothelial cells [7]. Hyperpermeability of the endothelium is one of the initial responses of vascular endothelial cells to a hyperglycemic environment [17]. NO produced by eNOS has been proven to play an essential role in protecting and maintaining vascular endothelial function [22,23]. As shown in Figure 4, NO production in AGEs-induced EA.hy926 cells in the model group (treated with 800 μg/mL AGEs) was significantly lower than that in the control group (treated with 800 μg/mL BSA; *p* < 0.05), and the NO contents in the model and control groups were 1.25 ± 0.04 μmol/L and 2.42 ± 0.02 μmol/L, respectively. Compared with the model group, A7G, I3R, and C3G can significantly increase NO production (*p* < 0.05). In particular, NO content in the I3R treatment at 40 μM was the highest at 2.37 ± 0.01 μmol/L, which presented no significant difference from that in the control group (*p* > 0.05), followed by C3G and then A7G. Several previous studies have found that maintaining NO bioavailability in vascular endothelial cells by dietary polyphenols is beneficial for improving endothelial dysfunction caused by diabetes [7,22,24]. Gerardi et al. reported that the gastrointestinal digestive products of wine lees contain a high content of flavonols that can regulate endothelial permeability by maintaining NO production [7]. Moreover, resveratrol can stimulate AMP-activated protein kinase to activate eNOS to produce NO, eventually improving vascular endothelial dysfunction in type 2 diabetes [22]. Similarly, Garcia et al. found that wine pomace seasoning rich in a variety of flavonoids significantly increased NO bioavailability in EA.hy926 cells in a high-glucose environment [24].

**Figure 5:**
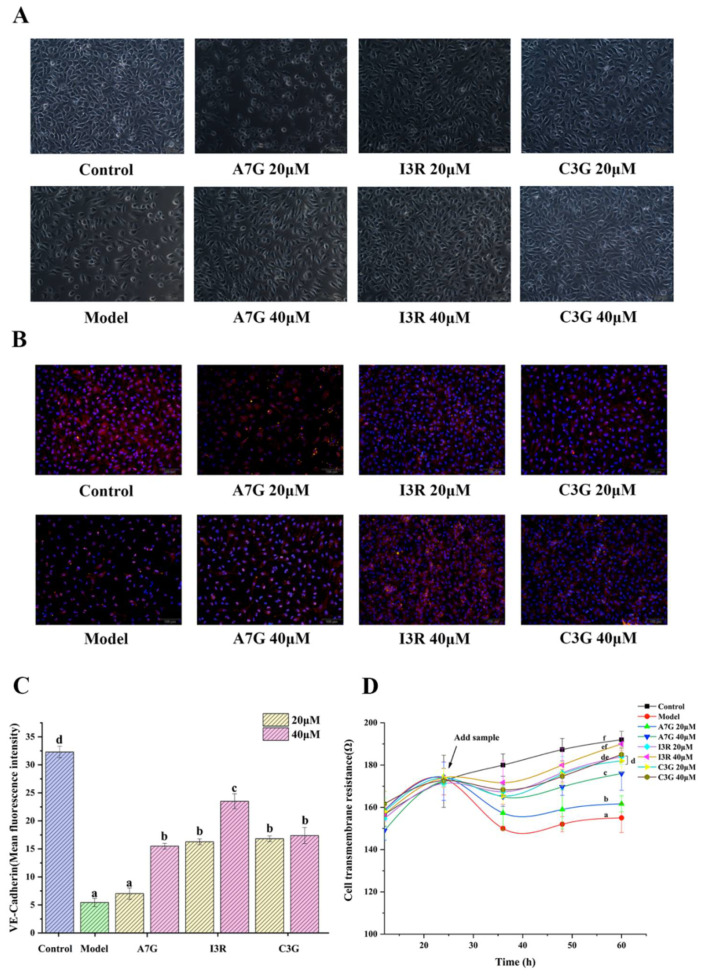
Protective effects of A7G, I3R, and C3G on endothelial functional barrier destruction induced by AGEs in EA.hy926 cells. (**A**) exhibits the cell morphology of EA.hy926 cells under a phase-contrast microscope under different sample processing conditions; (**B**) exhibits immunofluorescence staining of VE-cadherin (red) and nuclear DAPI (blue) of EA.hy926 cells under different sample treatments. (**C**) is the mean fluorescence intensity of VE-cadherin assessed using ImageJ. (**D**) is the TEER (transepithelial electrical resistance) value of EA.hy926 cells cultured on a Transwell plate that was continuously monitored over 60 h. All the values are expressed as mean ± SD. Means with different letters (a, b, c, d, e, f) are significant differences (*p* < 0.05).

The intercellular adhesion of endothelial cells primarily depends on the adhesion link, which is largely maintained by VE-cadherin. As a transmembrane protein, VE-cadherin has been proven to play an important role in maintaining the integrity of the endothelial barrier [17]. Under certain stress conditions, such as oxidative stress and hyperglycemia, the hyperphosphorylation of VE-cadherin is accelerated, leading to the dissolution of VE-cadherin and the destruction of adherent junctions between cells, eventually causing the exosmosis of macromolecules [25]. As shown in Figure 5A, intercellular space was evidently larger in the model group (treated with 800 μg/mL AGEs) than in the control group (treated with 800 μg/mL BSA). The change may be attributed to the structural changes of endothelial cells induced by AGEs, the endocytosis and dissolution of VE-cadherin in EA.hy926 endothelial cells, and the upregulation of cell adhesion molecules, increasing the monolayer permeability of endothelial cells [7,26]. However, different types and concentrations of flavonoids can evidently improve intercellular space to different degrees, except for A7G at 20 μM. The immunofluorescence results of the expression of VE-cadherin (red fluorescence) of vascular endothelial cells in Figure 5B,C further confirmed the phenomena observed in Figure 5A. Compared with that in the control group, the average fluorescence intensity of VE-cadherin in the model group was significantly lower (*p* < 0.05). Compared with the model group, the A7G (40 μM), I3G, and C3G treatment groups significantly increased the expression of VE-cadherin (red fluorescence) in vascular endothelial cells (*p* < 0.05). In particular, the average fluorescence intensity of VE-cadherin in the I3R (40 μM) treatment group was the highest among all the treatment groups (*p* < 0.05) and only lower than that of the control group. However, A7G (20 μM) exhibited no apparent improvement effect and no significant difference from the model group in accordance with average fluorescence intensity (*p* > 0.05). Suganya et al. reported that polyphenols can not only relieve oxidative stress but also protect the vascular barrier function, preventing diabetic vascular complications [26]. In accordance with the results of previous studies and the current research, AGEs can clearly increase vascular permeability by downregulating VE-cadherin to cause endothelial dysfunction, and then flavonoids may protect endothelial functional barriers by maintaining adhesion [27].

In addition, the vascular barrier function can also be characterized by measuring the TEER value of endothelial cells on the Transwell plate in vitro. TEER is a quantitative measurement for the integrity of the cell monolayer barrier [28]. Therefore, the TEER value of EA.hy926 cells was measured in the current study to investigate the protective effects of different types and concentrations of dietary flavonoids on the integrity of cell barrier with AGE induction. As shown in Figure 5D, the results indicated that the TEER value of the model group was significantly reduced compared with that of the control group (*p* < 0.05). Moreover, when the TEER value became stable, the resistance of the model group was only 155 ± 3.92 Ω, indicating that the integrity of the EA.hy926 cells’ monolayer was severely damaged by AGE induction. A7G, I3R, and C3G can significantly improve single-layer barrier integrity, and their TEER values were significantly higher than that of the model group at each tested time (*p* < 0.05). Among the treatment groups, I3R (40 μM) presented the strongest protection (*p* < 0.05), and no significant difference was observed between its TEER value (190 ± 3.06 Ω) and that of the control group (192 ± 2.12 Ω) at 60 h (*p* > 0.05). By contrast, A7G at 20 μM exhibited the weakest effect (*p* < 0.05). A previous report indicated that the low TEER and low expression of VE-cadherin were associated with increased membrane permeability in AGEs-induced EA.hy926 cells [29]. Lin et al. also determined that the major component of Polygonum multiflorum, namely, 2,3,5,4′-tetrahydroxystilbene-2-*O*-β-D-glucoside, can increase the TEER value of EA.hy926 endothelial cells, maintaining the stability of the endothelial cell barrier function [28].

### 3.4. Molecular Docking Study

Multiple intracellular signals transduction is activated when AGEs bind to RAGE, such as the generation of cellular oxidative stress and the subsequently evoked inflammation [30,31]. In addition, AGEs can cause the reorganization and redistribution of endothelial cytoskeletal actin, leading to increased endothelial cell permeability. PFN1 is considered the target molecule of endothelial cell damage induced by AGEs [6]. Some dietary flavonoids have been proven to reduce vascular complications caused by AGEs by selectively binding RAGE and PFN1 [32,33]. The binding degree of small molecule ligands to receptors can also reflect their protective effect to a certain extent and can be used to explain the difference in the protection of different small-molecule ligands [34]. In the current study, the interactions of the three dietary flavonoids (A7G, I3R, and C3G) with RAGE and PFN1 were investigated through molecular docking to explain the differences in the effects of those three dietary flavonoids on AGEs-induced inflammation and endothelial dysfunction from the chemical structure.

**Figure 6:**
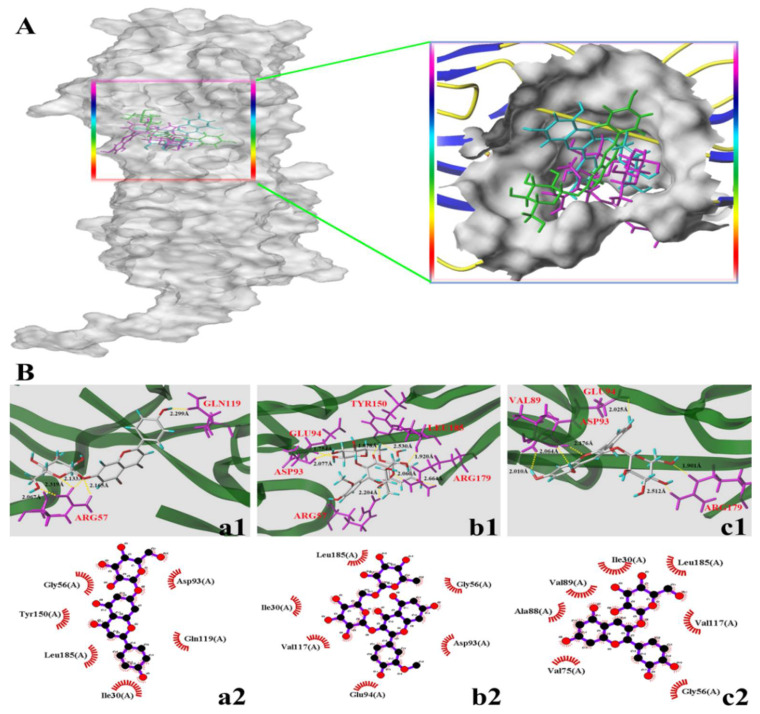
Molecular docking diagram of the compounds and the receptor of advanced glycation end products (RAGEs, PDB: 3CJJ). (**A**,**B**) in the figure represent the binding sites and binding conformations of small molecules in the RAGEs protein. The letters a, b, and c represent A7G, I3R, and C3G, respectively; a is green, b is purple, and c is cyan. Numbers 1–2 represent hydrogen bonding and hydrophobic interactions of small molecules and proteins, respectively.

The docking parameters and optimal docking conformation of the three dietary flavonoids with RAGE are presented in Table 1 and Figure 6, respectively. T-score is frequently used to determine the tight degree of binding between ligands and proteins [12]. Previous studies have reported that the binding degree of small-molecule ligands to receptors may be important indicators of the binding of AGEs and RAGE [34]. In accordance with the T-score (Table 1), the connection between C3G and RAGE was closer than that between I3R and RAGE and between A7G and RAGE. Figure 6A shows the different positions of the three types of flavonoids in the RAGE receptor, indicating that the three flavonoids are perfectly wrapped in the active pocket of the RAGE receptor (A7G: green; I3R: purple; and C3G: cyan). The hydrogen bonding and hydrophobic interaction between the three dietary flavonoids and the active pocket of the RAGE receptor are depicted in Figure 6B. A7G formed five hydrogen bonds with two amino acid residues of the RAGE active site (Arg57 and Gln119) and established hydrophobic interactions with six amino acid residues. I3R formed eight hydrogen bonds with six amino acid residues (Arg57, Asp93, Glu94, Tyr150, Arg179, and Leu185) and hydrophobic interactions with six amino acids. C3G formed six hydrogen bonds with four amino acid residues (Val89, Asp93, Glu94, and Arg179) and hydrophobic interactions with seven amino acid residues. The mean hydrogen bond distances of A7G, I3R, and C3G were 2.1966, 2.1359, and 2.220 Å, respectively. A previous study showed that the binding of ligand and RAGE, such as flavonoids–RAGE, to block AGEs–RAGE combination is an effective approach for alleviating AGEs-induced oxidative damage and inflammation [35,36]. Kajal et al. reported that the extract of *Coreandrum sativum* L. seeds, which is rich in flavonoids, can form hydrogen bonds and establish hydrophobic interactions with RAGE residues to inhibit diabetic complications [37]. Molecular docking results in the present work may further explain to a certain extent the mechanisms and differences among the three dietary flavonoids in inhibiting AGEs-induced inflammation from another perspective.

**Figure 7:**
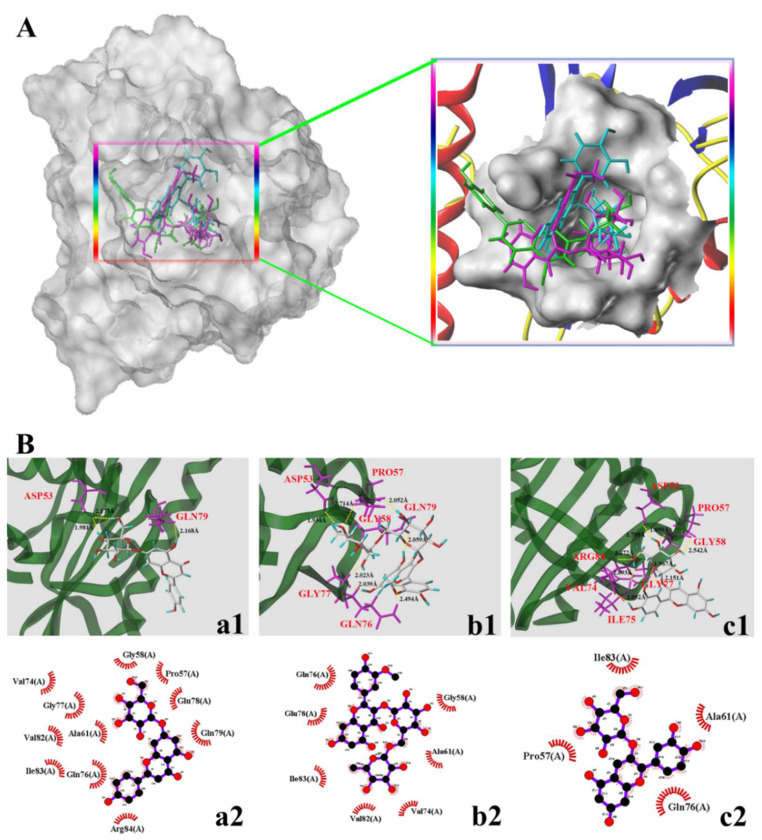
Molecular docking diagram of the compounds and the receptor of profilin-1 (PNF1, PDB: 3NUL). (**A**,**B**) in the figure represent the binding sites and binding conformations of small molecules in the PNF1 protein. The letters a, b, and c represent A7G, I3R, and C3G, respectively; a is green, b is purple, and c is cyan. Numbers 1–2 represent hydrogen bonding and hydrophobic interactions of small molecules and proteins, respectively.

Table 2 and Figure 7 provide the docking parameters and optimal docking conformation of the three dietary flavonoids with PFN1. From the T-score values in Table 2, I3R connected more tightly with PFN1 than C3G or A7G. Similarly, Figure 7A shows the different positions of the three types of flavonoids in the PFN1 receptor and the active pocket wrapped securely around them (A7G: green; I3R: purple; and C3G: cyan). The hydrogen bonding and hydrophobic interaction between the three dietary flavonoids and the active pocket of the PFN1 receptor are presented in Figure 7B. A7G formed three hydrogen bonds with Asp53 and Gln79 and established hydrophobic interactions with 11 amino acid residues. The average distance of the hydrogen bonds was 2.108 Å. I3R formed seven hydrogen bonds with the six amino acid residues in the active site of PFN1, namely Asp53, Pro57, GLy58, Gln76, Gly77, and Gln79, and established hydrophobic interactions with six amino acids. The average distance of the hydrogen bonds was 2.188 Å. C3G formed eight hydrogen bonds with seven amino acid residues (Asp53, Pro57, Gly58, Val74, Ile75, Gly77, and Arg84) and established hydrophobic interactions with four amino acid residues. The average hydrogen bond distances of C3G were 2.096. The major role of PFN1 has been reported as binding actin monomers to form actin cytoskeleton. Such a process will affect the integrity and endocytosis of the receptor scaffold when it is bound by a ligand [33]. A previous study pointed out that flavonoids may prevent or improve the reorganization and redistribution of the endothelial cytoskeleton by binding to PFN1 [6]. This finding is consistent with the results of the present work.

## 4. Conclusions

**Figure 8:**
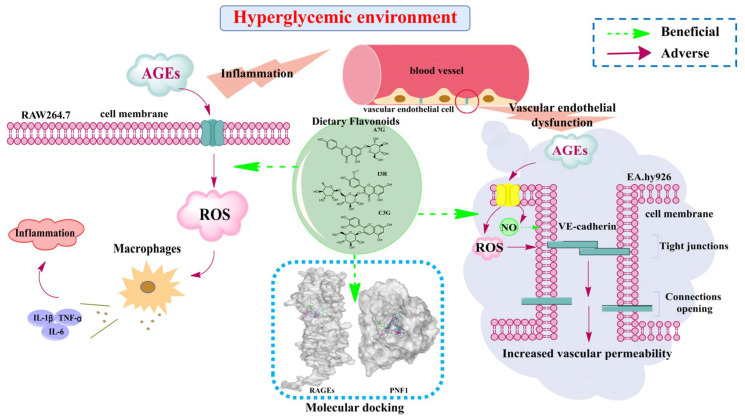
Research contents and mechanism of the effects of three dietary flavonoids (A7G, I3R, and C3G) on inflammation and vascular endothelial dysfunction caused by advanced glycation end products (AGEs).

In the current study, the effects of three dietary flavonoids, namely A7G, I3R, and C3G, on inflammation and vascular endothelial dysfunction caused by advanced glycation end products (AGEs) were comparatively investigated as outlined in Figure 8. Results showed that the preventive effects of those three dietary flavonoids on inflammation and vascular injury caused by AGEs were substantially dependent on their structures and concentrations. In AGEs-induced RAW264.7 cells, I3R (40 μM) demonstrated the strongest inhibitory effect on intracellular ROS, followed by C3G, with A7G as the weakest. However, C3G exhibited the strongest inhibitory effects on inflammatory cytokines (TNF-α, IL-1β, and IL-6), followed by I3R, and A7G. Molecular docking results have also shown that the T-score of the combination between C3G and RAGE was the highest, indicating the closest binding. In AGEs-induced EA.hy926 cells, I3R (40 μM) presented the strongest bioactivities on the inhibition of intracellular ROS, increase of NO production, and maintenance of endothelial cell permeability, followed by C3G and A7G. The molecular docking results also showed that I3R can connect more closely with PFN1 and achieved the highest T-score. The results obtained in the current study suggest that food abundant in the three flavonoids, particularly I3R and C3G, may effectively prevent or ameliorate AGEs-induced vascular complications. The results of the current work may provide new knowledge and helpful suggestions regarding the benefits of dietary flavonoids in diabetic vascular complications. In the future, a series of in-depth and comprehensive studies could be performed to fully illuminate the underlying mechanisms of different dietary flavonoids on alleviating AGEs-induced inflammation and vascular endothelial dysfunction.

## Figures and Tables

**Table 1:** Docking parameters, hydrophobic interactions, and hydrogen bonds observed between flavonoids and the receptor of advanced glycation end products (RAGEs) from molecular docking simulation analysis. The hydrophobic interactions between flavonoids and RAGEs were analyzed by Ligplot^+^ software according to the molecular docking results obtained from SYBYL software. A7G: apigenin-7-*O*-glucoside, I3R: isorhamnetin-3-*O*-rutinoside, C3G: cyanidin-3-*O*-glucoside, PMF: Potential of Mean Force, CHEM: Chemical, Arg: Arginine, Asp: Asparticacid, Ala: Alanine, Gln: Glutamine, Glu: Glutamicacid, Gly: Glycine, Ile: Isoleucine, Leu: Leucine, Tyr: Tyrosine, Val: Valine.

	A7G	I3R	C3G
C-score	4	4	4
T-score	5.0281	5.2227	7.349
D-score	−90.4449	−125.569	−143.8509
PMF-score	−46.7436	−77.7488	−51.0954
G-score	−113.5269	−114.4509	−192.7999
CHEM-score	−12.429	−14.0453	−22.6174
Number of hydrogen bonds	5	8	6
Amino acid residues involved in hydrogen bonds	Arg57, Gln119	Arg57, Asp93, Glu94, Tyr150, Arg179, Leu185	Val89, Asp93, Glu94, Arg179
Number of hydrophobic interactions	6	6	7
Amino acid residues involved in hydrophobic interactions	Ile30, Gly56, Asp93, Gln119, Tyr150, Leu185,	Ile30, Gly56, Asp93, Glu94, Val117, Leu185	Ile30, Gly56, Val75, Ala88, Val89, Val117, Leu185,
Average distance (Å)	2.1966	2.1359	2.220

**Table 2:** Docking parameters, hydrophobic interactions, and hydrogen bonds observed between flavonoids and the receptor of profilin-1 (PFN1) from molecular docking simulation analysis. The hydrophobic interactions between flavonoids and PFN1 were analyzed by Ligplot^+^ software according to the molecular docking results obtained from SYBYL software. A7G: apigenin-7-*O*-glucoside, I3R: isorhamnetin-3-*O*-rutinoside, C3G: cyanidin-3-*O*-glucoside, PMF: Potential of Mean Force, CHEM: Chemical, Arg: Arginine, Asp: Asparticacid, Ala: Alanine, Gln: Glutamine, Glu: Glutamicacid, Gly: Glycine, Ile: Isoleucine, Pro: Proline, Val: Valine.

	A7G	I3R	G3G
C-score	5	4	5
T-score	3.9599	4.4235	3.6886
D-score	−103.0791	−107.758	−109.7659
PMF-score	−10.9616	−2.636	17.8642
G-score	−152.0601	−105.9407	−126.2047
CHEM-score	−12.8453	−17.9643	−21.6537
Number of hydrogen bonds	3	7	8
Amino acid residues involved in hydrogen bonds	Asp53, Gln79	Asp53, Pro57, Gly58, Gln76, Gly77, Gln79	Asp53, Pro57, Gly58, Val74, Ile75, Gly77, Arg84
Number of hydrophobic interactions	11	6	4
Amino acid residues involved in hydrophobic interactions	Gly58, Pro57, Ala61, Val74, Gln76, Gly77, Glu78, Gln79, Val82, Ile83, Arg84	Gly58, Ala61, Val74, Gln76, Glu78, Ile83	Pro57, Ala61, Gln76, Ile83
Average distance (Å)	2.108	2.188	2.096

## Data Availability

The data that support the findings of this study are available from the corresponding author upon reasonable request.

## Supplementary Materials

The following supporting information can be downloaded at: https://www.mdpi.com/article/10.3390/nu14051026/s1, Figure S1: The AGEs-specific fluorescence, which was measured at 370 nm excitation and 440 nm emission wavelengths by using a SpectraMax M5 microplate.
