# Supplementary material for: Dietary Flavonoids Alleviate Inflammation and Vascular Endothelial Barrier Dysfunction Induced by Advanced Glycation End Products In Vitro
Source: Nutrients. 2022 Feb 28;14(5):1026. doi: 10.3390/nu14051026 (PMC8912803; doi:10.3390/nu14051026)
Supplement: Supplementary file 1 [file nutrients-14-01026-s001.zip › nutrients-1567773-supplementary.pdf]

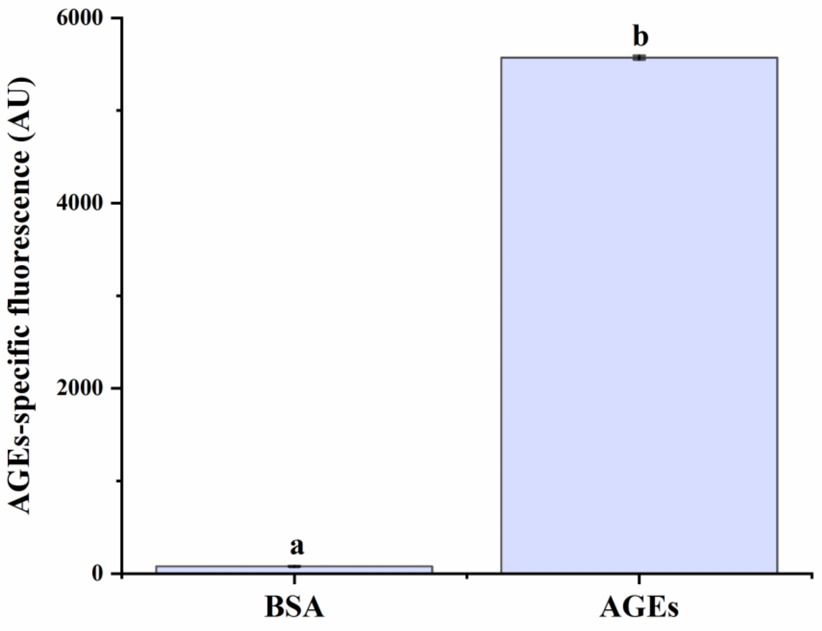

Figure S1. The AGEs-specific fluorescence, which were measured at 370 nm excitation and 440 nm emission wavelengths by using a SpectraMax M5 microplate.
